# Personalized statistical modeling of soft tissue structures in the knee

**DOI:** 10.3389/fbioe.2023.1055860

**Published:** 2023-03-08

**Authors:** A. Van Oevelen, K. Duquesne, M. Peiffer, J. Grammens, A. Burssens, A. Chevalier, G. Steenackers, J. Victor, E. Audenaert

**Affiliations:** ^1^ Department of Orthopedic Surgery and Traumatology, Ghent University Hospital, Ghent, Belgium; ^2^ Department of Human Structure and Repair, Ghent University, Ghent, Belgium; ^3^ InViLab research group, Department of Electromechanics, University of Antwerp, Antwerp, Belgium; ^4^ Antwerp Surgical Training, Anatomy and Research Centre (ASTARC), University of Antwerp, Wilrijk, Belgium; ^5^ Imec-VisionLab, Department of Physics, University of Antwerp, Antwerp, Belgium; ^6^ Cosys-Lab research group, Department of Electromechanics, University of Antwerp, Antwerp, Belgium; ^7^ Department of Trauma and Orthopedics, Addenbrooke’s Hospital, Cambridge University Hospitals NHS Foundation Trust, Cambridge, United Kingdom

**Keywords:** statistical shape modeling, soft-tissue modeling, computational modeling, personalized medicine, knee joint

## Abstract

**Background and Objective:** As *in vivo* measurements of knee joint contact forces remain challenging, computational musculoskeletal modeling has been popularized as an encouraging solution for non-invasive estimation of joint mechanical loading. Computational musculoskeletal modeling typically relies on laborious manual segmentation as it requires reliable osseous and soft tissue geometry. To improve on feasibility and accuracy of patient-specific geometry predictions, a generic computational approach that can easily be scaled, morphed and fitted to patient-specific knee joint anatomy is presented.

**Methods:** A personalized prediction algorithm was established to derive soft tissue geometry of the knee, originating solely from skeletal anatomy. Based on a MRI dataset (*n* = 53), manual identification of soft-tissue anatomy and landmarks served as input for our model by use of geometric morphometrics. Topographic distance maps were generated for cartilage thickness predictions. Meniscal modeling relied on wrapping a triangular geometry with varying height and width from the anterior to the posterior root. Elastic mesh wrapping was applied for ligamentous and patellar tendon path modeling. Leave-one-out validation experiments were conducted for accuracy assessment.

**Results:** The Root Mean Square Error (RMSE) for the cartilage layers of the medial tibial plateau, the lateral tibial plateau, the femur and the patella equaled respectively 0.32 mm (range 0.14–0.48), 0.35 mm (range 0.16–0.53), 0.39 mm (range 0.15–0.80) and 0.75 mm (range 0.16–1.11). Similarly, the RMSE equaled respectively 1.16 mm (range 0.99–1.59), 0.91 mm (0.75–1.33), 2.93 mm (range 1.85–4.66) and 2.04 mm (1.88–3.29), calculated over the course of the anterior cruciate ligament, posterior cruciate ligament, the medial and the lateral meniscus.

**Conclusion:** A methodological workflow is presented for patient-specific, morphological knee joint modeling that avoids laborious segmentation. By allowing to accurately predict personalized geometry this method has the potential for generating large (virtual) sample sizes applicable for biomechanical research and improving personalized, computer-assisted medicine.

## 1 Introduction

Osteoarthritis (OA) affects almost one out of 4 people globally and represents one of the fastest growing socio-economic burdens in the world (Hunter et al., 2020; Boer et al., 2021). Knee OA constitutes 83% of the global disease burden for OA (Vos et al., 2012). Although highly prevalent, researchers are only at dawn of unravelling the complex interaction between both biomechanical and systemic factors triggering disease onset and progression (Sharma et al., 2010).

Methodologies to accurately measure *in vivo* joint contact forces acting on the knee and to analyze soft tissue functioning are currently lacking and hamper research progression. A valuable approach that is increasingly being adopted, involves computational musculoskeletal modeling to indirectly estimate joint mechanics. This method allows for non-invasive estimation of joint loading distribution while improving insight in intersubjective anatomical variance when repeatedly performed. While it has been previously shown that the results of these models rely strongly on accurate anatomical information, generation of such input structures is generally deducted from manual segmentation of Computed Tomography (CT) and/or Magnetic Resonance Imaging (MRI) (Marra et al., 2015; Kang et al., 2017). However, repeated laborious manual segmentations remain a substantial bottleneck of the personalized musculoskeletal modeling workflow. In addition, manual segmentations contribute to a higher rate of observer-related inaccuracies (Seim et al., 2008; Bae et al., 2009). Furthermore, the frequent use of MRI is complicated by a high cost and a low availability (Bae et al., 2009). To date, these limitations impede the bench to bedside translation and routine use in clinical practice.

An emerging approach to mitigate this problem is the combination of computational musculoskeletal modeling with statical shape analysis (van Houcke et al., 2020a; Pascoletti et al., 2021). Aiming to bypass the aforementioned restrictions, Audenaert and colleagues developed a validated pipeline for semi-automated shape model-based segmentation of the lower limb based on computed tomography (CT) imaging (van Haver et al., 2014a; Audenaert et al., 2019a). Next, Van Houcke and colleagues tackled the issue of personalized cartilage layer geometry prediction by the development of cartilage thickness maps based on a training MRI dataset and by building on the features of simplicity and anatomical correspondence of geometric morphometrics. Femoroacetabular cartilage geometry was thus estimated according to hip joint morphometrics, avoiding the manual segmentation inaccuracies and optimizing time-efficiency (van Houcke et al., 2020a). For the inclusion of muscle and tendon paths, Audenaert et al. overcame the hurdle of modeling deformable soft tissue utilizing discrete elements rigid body spring models, again relying on geometric morphometrics for the prediction of origin and insertion (Audenaert and Audenaert, 2008; Audenaert et al., 2019b).

The combined methodology to describe bone, cartilage and soft tissue at a population wide level was recently adopted for the ankle joint by (Peiffer et al., 2022a). In this study, the techniques described by Van Houcke et al. and Audenaert et al. were combined for estimation of cartilage topography of the tibiotalar joint and inclusion of the main ankle ligament paths (Audenaert et al., 2019b; van Houcke et al., 2020a; Peiffer et al., 2022a). However, no similar advancements have been made for the knee joint. Van Dijck and colleagues developed a statistical shape model (SSM) based on 524 knee joint MRI’s to predict cartilage thickness and localization in the tibiofemoral joint. This model, however, lacks inclusion of patellar bone and cartilage layer as well as the menisci and the cruciate ligaments (van Dijck et al., 2018).

The recent introduction of SSM allows for efficient population-wide analysis of shape as variance is compactly modeled and is an established tool for medical image segmentation. Being able to simulate large populations, the use of SSM improves understanding of disease models and injury biomechanics. Furthermore, the use of SSM enables patient-specific modeling which facilitates the introduction of individualized medicine in the clinical practice (Nauwelaers et al., 2021). However, an inclusive, patient-specific computational knee joint model, minimally relying on manual segmentation is currently lacking.

The aim of this study is to develop a generic computational model that can easily be scaled, morphed and fitted to patient-specific knee joint anatomy, avoiding laborious segmentation tasks and improving accuracy of patient-specific geometry predictions. This study builds further on in-house available expertise regarding statistical shape modeling and soft tissue wrapping methodology (Audenaert et al., 2019b; van Houcke et al., 2020a; Peiffer et al., 2022a). The objectives of the current study are: 1. Patient-specific prediction of the cartilage layer of the tibiofemoral and the patellofemoral joint, 2. Prediction of the anatomy of the anterior and posterior cruciate ligament, main knee ligaments and patellar tendon, 3. Prediction of static meniscal anatomy, and 4. Validation of patient-specific soft tissue prediction.

## 2 Materials and methods

### 2.1 Data collection

Two distinct imaging databases were used for the shape modeling workflow. In particular, a first dataset consisted of CT images, adopted for the SSM development and the description of osteology, whereas soft tissue features were derived from a second dataset, consisting of MR images.

For the description and parameterization of the osseous structures, a total of 311 bilateral lower limb CT scans (training sample *n* = 622) were derived of 181 male and 130 female non-arthritic subjects. The average age of males and females was respectively 67.8 (±10.8) and 69 (±13.3) years. Each scan contained an average of 1864 slices with a pixel size 0.575 mm–0.975 mm. This imaging data were previously used in the development of an articulated skeletal SSM of the lower limb, including the knee joint (Audenaert et al., 2019a). A detailed description of the articulated skeletal SSM generation and validation in terms of specificity, compactness, generalizability, accuracy and population coverage was previously published (Audenaert et al., 2019a).

For the description and parameterization of soft tissue structures, the extensive MRI database built by Van Hoecke et al. was used (Van Hoecke et al., 2020b). This database consisted of 53 young and healthy Caucasian men who underwent dedicated, high resolution series of hip, knee and ankle joints in an unloaded position. The dataset contained healthy Caucasian men aged between 17 and 25 years who were not overweight with a mean total body length of 181.79 cm. Dedicated hip, knee and ankle scans were taken using a Siemens^®^ 3 Tesla MRI with a pixel size of 0.469 mm–0.469 mm and a slice thickness of 0.5 mm (van Houcke et al., 2020b; de Roeck et al., 2020). These dedicated scans were then stitched using Materialise’s Interactive Medical Image Control System (Mimics^®^ v21.0, Materialise, Leuven, Belgium) in the formation of an overview, full lower limb scan. Further details on data collection and imaging acquisition were previously published (van Houcke et al., 2020b).

Subjects included in both studies provided written informed consent. An ethics committee of the Ghent University Hospital (Belgium) approved both investigations.

### 2.2 MRI segmentation, landmark identification and definition of structural features

MR scan data were exported as Digital Imaging and Communications in Medicine (DICOM) files and subsequently imported in Mimics^®^. Osseous and cartilage anatomy was extracted from all 53 cases (see section 2.2.1 and 2.2.2). Identification of dedicated landmarks and structural features (e.g., thickness, height and width) used in ligament, patellar tendon and meniscal anatomy prediction was performed on 10 cases. The used cases were randomly selected from the complete dataset. All calculations were completed in Matlab by using both custom-made Matlab^®^ scripts and the Matlab^®^ plugin in the Mimics software, and performed on a Dell Precision 5560 Laptop (Intel Core i9 -11950H, 64 GB RAM, 64 bit).

#### 2.2.1 Segmented osseous anatomy

The osseous anatomy was derived from the overviewing full lower limb MRI scans, relying on SSM-based semi-automated image segmentation. First, 300 points were manually determined, randomly distributed over the cortical edges of the structures for which segmentation is required (e.g., the femoral bone, the patellar bone and the combined tibial-fibular bone). Second, the SSM of the corresponding structure was fitted (van Haver et al., 2014a; van Haver et al., 2014b; Audenaert et al., 2019a). For fitting, a total of 50 principal components was retained, resulting in a cumulative explained variance of 99.55%, 96.60% and 99.19% for respectively the femur, the patella and the combined tibia-fibula. These target meshes were thus dense corresponding surface geometries provided by means of quasi-isometric triangulated meshes consisting out of 21097, 3825 and 39197 vertices and 42188, 7646 and 78386 faces for respectively the femur, the patella and the combined tibia with fibula. Uniformly distributing the total of vertices over the osseous structure, the average length of the triangle edges equaled respectively 1.77 mm, 1.05 mm and 1.24 mm for the femur, the patella and the combined tibia with fibula. Audenaert and colleagues evaluated the accuracy of SSM-based segmentation against manual segmentation based on the Average Surface Distance (ASD) and the Hausdorff Distance (HD). The ASD equaled 0.65 mm (SD 0.10 mm), 0.63 mm (SD 0.11 mm) and 0.76 mm (SD 0.18 mm) for respectively the femoral, tibial and fibular bone. The HD equaled respectively 4.79 mm (SD 2.39 mm), 4.07 mm (SD 2.15 mm) and 3.76 mm (SD 1.17 mm). Based on the proven generalizability of the model, accurate SSM-based segmentation was obtained (Audenaert et al., 2019a). From these triangulated meshes, the distal femur, the proximal tibia and the patella were isolated and imported for further use in the high resolution series of the knee in Mimics^®^.

#### 2.2.2 Segmented cartilage anatomy

For all 53 cases, the cartilage layers of distal femur, proximal tibia and patella were manually segmented on the dedicated high resolution knee series. The contour editing tool in Mimics^®^ was used to deform the uniform osseous meshes of the femur, the patella and the combined tibia-fibula, to no longer solely delineating the osseous cortex but additionally comprising the cartilage layer. The Mimics^®^ contour editing tool applied a distance-based Gaussian deformation, a type of free-form deformation, to provide a smooth contour edit and to control locality. As such, it allowed for point correspondence between the osseous mesh and the deformed mesh comprising the cartilage layer (Yoshida et al., 2002).

#### 2.2.3 Landmarks defining ligamentous and patellar tendon anatomy

Ligamentous and patellar tendon origin and insertion sites were manually selected. The selection was supported and guided by the anatomical reference of Laprade et al. (LaPrade and Engebretsen, 2007; James et al., 2015). To minimize the error related to manual landmark identification, landmarks were first localized on MRI in 10 cases. Subsequently, the location of the origin and insertion points was established in relation to the bony surfaces of femur, tibia and patella as nearest neighboring points were derived. Lastly, the average origin and insertion was determined and annotated on a reference template mesh. Non-rigid surface registration of the reference template towards the SSM allowed for indices-based landmark transfer within the osseous shape model while maintaining anatomical correspondence (van Haver et al., 2014b). Ligamentous thicknesses, later required for ligament modeling, were obtained from literature (Nomura et al., 2005; Wilson et al., 2012; Hedderwick et al., 2017; Ariel de Lima et al., 2019; Atkinson et al., 2022). The described ligaments included the Medial Patellofemoral Ligament (MPFL), the Lateral Patellofemoral Ligament (LPFL), the two strands of the superficial Medial Collateral Ligament (sMCL) (e.g., an anterior and posterior bundle), the Lateral Collateral Ligament (LCL), the Anterolateral Ligament (ALL), the Posterior Oblique Ligament (POL) and the Oblique Popliteal Ligament (OPL).

#### 2.2.4 Landmarks and structural features defining cruciate ligament anatomy

Similarly, origin and insertion sites of the anterior (ACL) and posterior (PCL) cruciate ligament were manually selected, averaged and annotated on a reference template mesh, allowing non rigid registration towards the SSM. Additionally, the thickness of the ACL and PCL over their respective course from origin to insertion was measured for every 10 slices on MRI, to describe local variation in their respective radius.

#### 2.2.5 Landmarks and structural features defining meniscal anatomy

**FIGURE 1:**
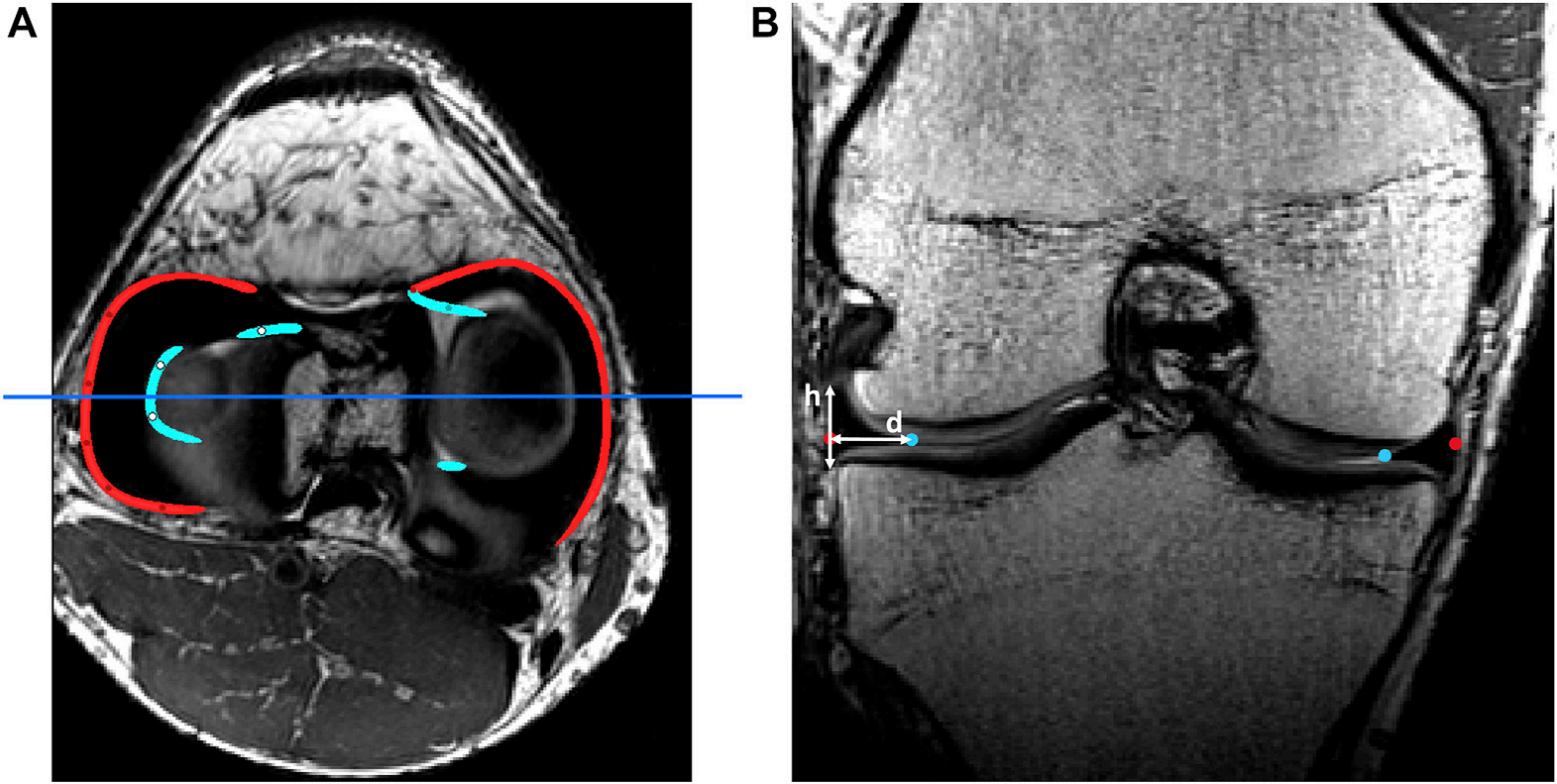
**(A)** Axial view of the knee joint on MRI with manually detected outer rims (red) and inner rims (light blue). The dark blue line represents the slice of the coronal view. **(B)** Coronal view of the knee joint on MRI. The height (h) and width (d) of the meniscus were measured as shown. This process was repeated over the course of the outer rim.

Similar to ligament and patellar tendon inclusion, the anterior and posterior root of the medial and lateral meniscus were subsequently manually selected, averaged and determined in relation to the tibia anatomy. Following, corresponding nearest indices values were derived to allow for landmark transfer within the shape model. An inner and outer rim was generated based on manually selected points on MRI, using the spline generation tool incorporated in Mimics^®^. The meniscal height and width was repeatedly measured from origin to insertion to describe the triangular geometry of the meniscus and local variation herein. For randomly selected points distributed over the outer rim, the meniscal height was measured and the meniscal width was defined as the Euclidean distance between the inner and outer meniscal rim. A detailed description of this process is described in Figure 1.

### 2.3 Workflow for subject-specific, soft tissue prediction

#### 2.3.1 Cartilage thickness prediction

**FIGURE 2:**
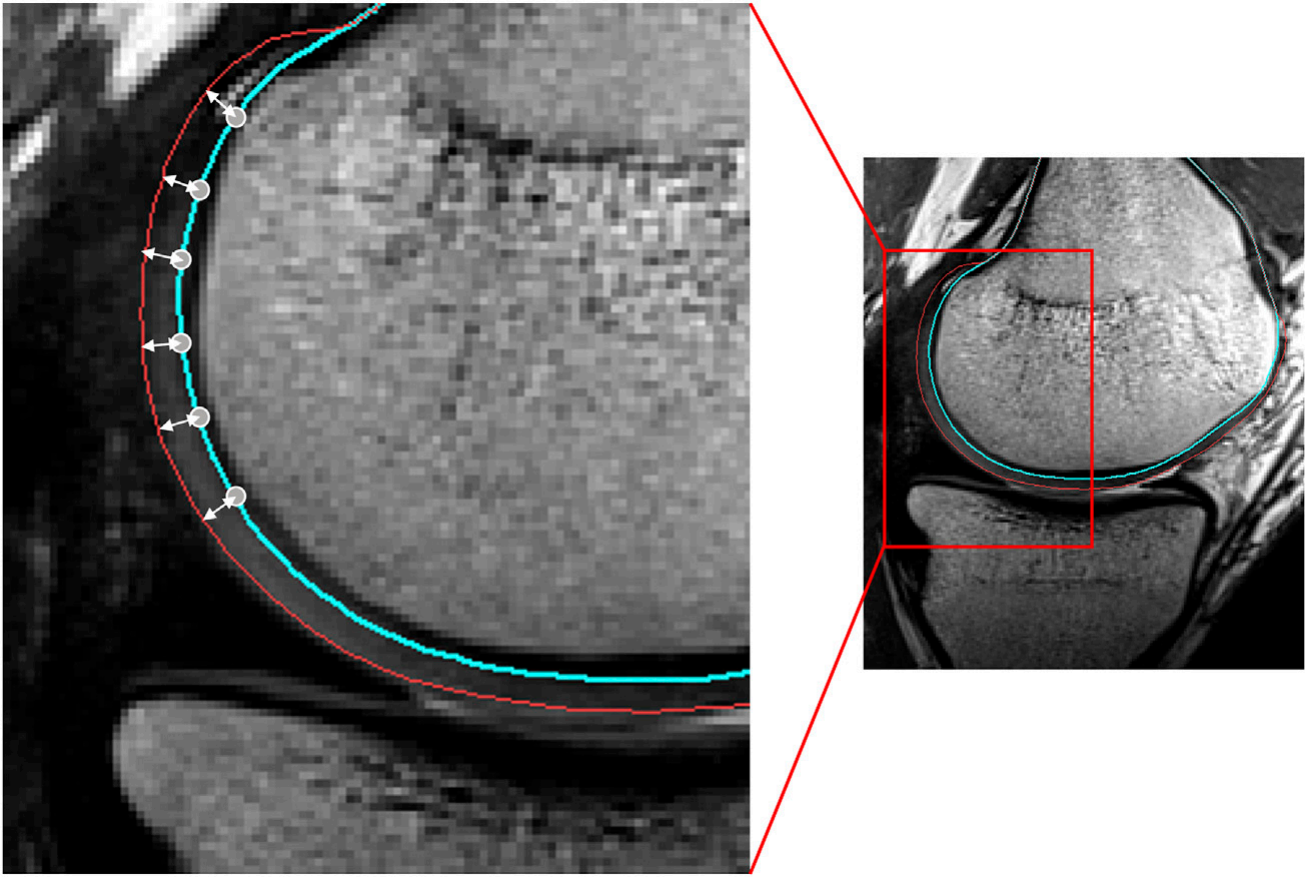
Sagittal view of the femoral condyle on MRI. The node-specific cartilage thickness was defined as the distance of the vertex (located on the blue line) to the cartilage surface (red), along the vertex normal.

To determine the location of the cartilage layer, the previously developed meshes of the osseous structures and the ones including the cartilage layers were easily compared, as correspondence was maintained following Gaussian-based contour editing in Mimics^®^. A total of 2221 vertices, 872 vertices, 875 vertices and 1572 vertices were identified for respectively the distal femoral bone, the medial tibia plateau, the lateral tibia plateau and the patellar bone. For every case, the node-specific cartilage thickness was defined as the distance of the subchondral bone to the cartilage surface, along the surface normal. To smoothly attach the cartilage to the bone, the distances at the edge vertices were adjusted to zero. As such, 53 case-specific cartilage thickness maps were generated (Figure 2).

These thickness maps were averaged to develop a mean cartilage thickness map. The cartilage geometry of any new shape was then predicted by projecting the vertices, part of the articular surface, along the direction of their normal over their corresponding distance, extracted from the mean distance map. As previous research has already demonstrated the correlation between osseous size and thickness of the cartilage layers, the dedicated distance maps were scaled according to the femoral length to account for size differences between cases (Rissech et al., 2013; van Dijck et al., 2018; Schneider et al., 2022). Thus, the cartilage thickness does not solely depend on the morphology of the underlying bone.

#### 2.3.2 Meniscal anatomy prediction

Both menisci were modeled as mobile, elastic structures accommodating to the shape of the femoral condyles and their variable position relative to the tibia. The geometry of both menisci was numerically simplified as triangular with varying height and width. The previously derived height and width measures were averaged and plotted against the relative outer length of the meniscus (Figure 1). Polynomial functions of increasing order were fitted and compared to the ground truth, an over-fitted 20th degree polynomial, by means of the Root Mean Square Error (RMSE). Evolution in the RMSE for increasing order of the polynomial enabled the detection of the optimal degree of polynomial fitting. Based on the coefficients extracted from the optimal degree polynomial function, meniscal height and width were calculated over the course of the meniscus from anterior to posterior root. As such, this method allowed for description of regional variation in meniscal triangular geometry.

**FIGURE 3:**
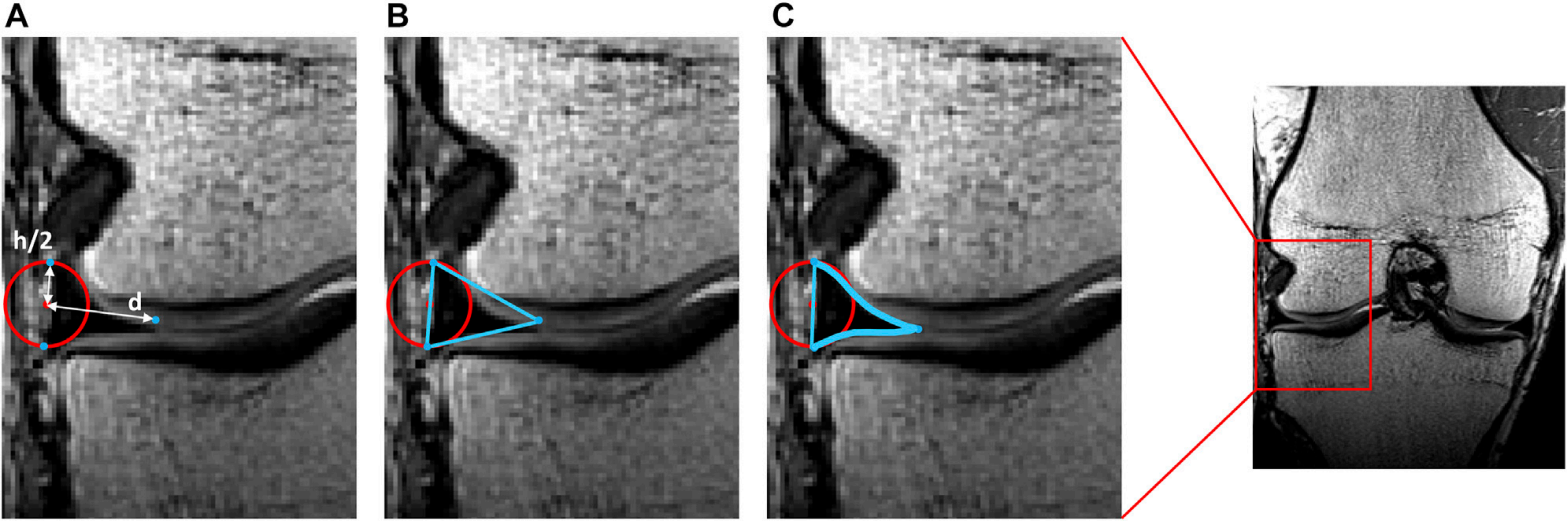
Representation of meniscal modeling. **(A)** A tube was wrapped elastically from the anterior to the posterior horn enforcing an offset of half the meniscal height (h/2). A varying distance (d) is extracted for every node. **(B)** Triangles are formed by interconnecting the three points per node. **(C)** Following correction for local penetration, the edges of the meniscus adapt to fit in between cartilage layers.

**FIGURE 4:**
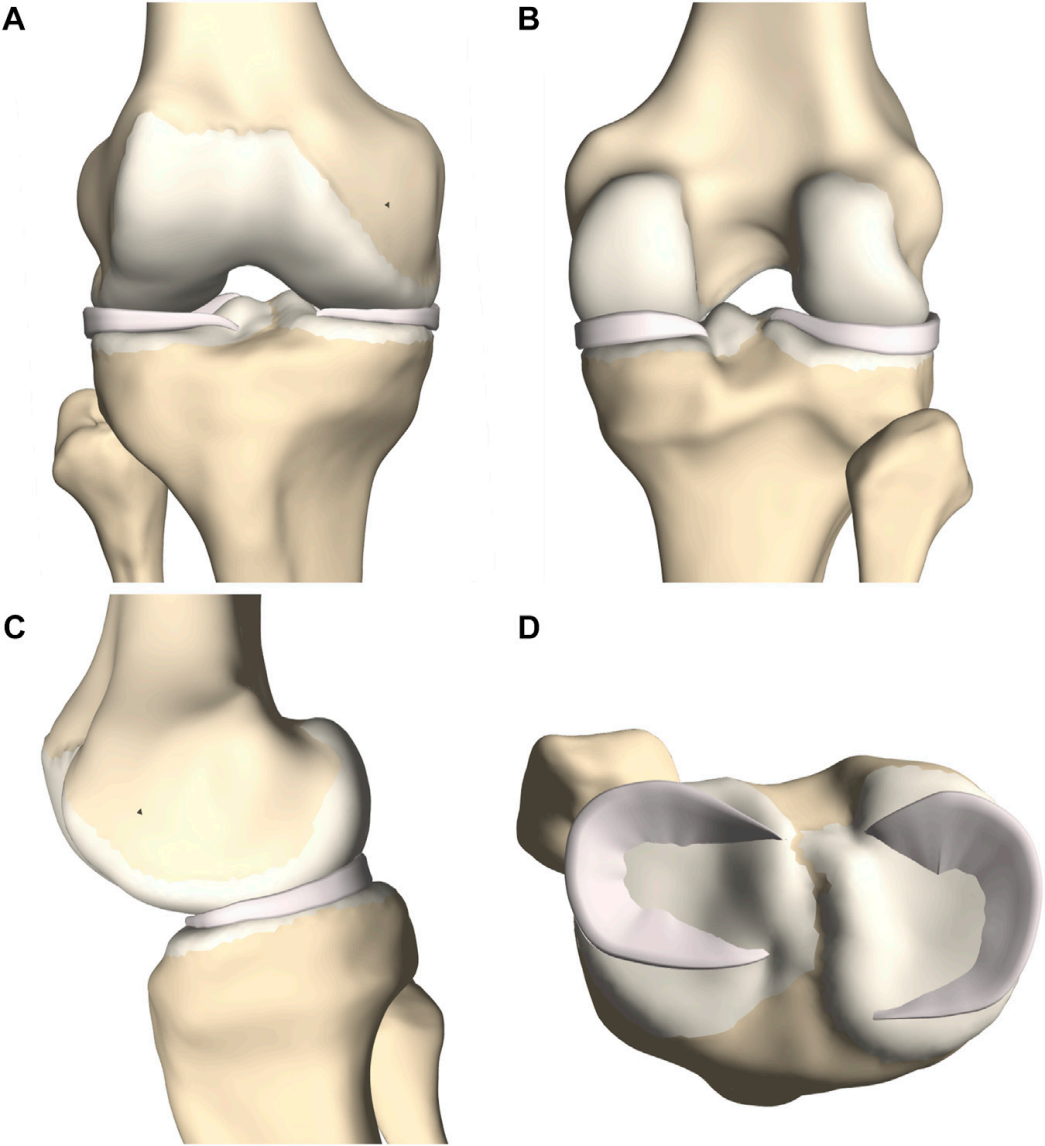
**(A)** Frontal, **(B)** posterior and **(C)** medial view of femoral and tibial bone (yellow) with predicted cartilage layers (white) and menisci (pink). **(D)** Axial view on the tibial plateau (yellow), with predicted cartilage layers (white) and menisci (pink).

First and relying on previous work, a tube was elastically wrapped from the anterior to the posterior horn enforcing an offset equal to half the meniscal height. Tube formation, as described by Audenaert and colleagues, was based on a generalized cylinder model. Over the path of a spline, connecting the anterior to the posterior meniscal root, the point-dependent offset was imposed to avoid osseous and cartilage penetration and thus force the tube to wrap around the femoral condyle (Audenaert et al., 2009; Audenaert et al., 2019b; Peiffer et al., 2022a). Second, according to the calculated path and at equidistant interval, triangles were defined with height and width derived from the above described polynomial estimates, with the width projected towards the meniscal center and the height orthogonal to this. The triangles, generated by interconnecting the three projections per node were then logically arranged to form a 3D mesh. Third, to correct for local penetration and to fit meniscal geometry between femoral and tibial cartilage, local meniscal morphometry was adjusted by projecting penetrating meniscal nodes on the outer osseous–cartilage surface (Figures 3, 4).

#### 2.3.3 Ligament and patellar tendon anatomy prediction

Ligament geometry and path prediction was based on a custom-made mesh wrapping algorithm as previously described by (Audenaert et al., 2009; Audenaert et al., 2019b; Peiffer et al., 2022a). This mesh wrapping algorithm was based on path prediction of the psoas anatomy as described by Audenaert et al. A finite number of springs formed an elastic membrane that was iteratively released to progressively minimize the potential energy while not permitting penetration of underlying structures (Audenaert et al., 2019b). Peiffer et al. converted this technique towards ligamentous modeling as elastic line segments rather than membranes connected ligamentous origins and insertions. Again, by progressively releasing the elastic segment, the potential energy was minimized without permitting penetration of adjacent structures. Penetrating nodes were returned to the closest point on the penetrated surface. Applying this technique for knee soft tissue modeling, knee ligaments were wrapped around the osseous-cartilage–meniscus meshes while surface penetration was impeded. Following, a flat mesh was formed by interconnecting the nodes (Peiffer et al., 2022a). Lastly, a volume was added to the ligament description by assigning a single, ligament-specific thickness over the course of the ligament. This thickness was based on previously published cadaveric studies and MRI measurements (Table 1) (Nomura et al., 2005; Wilson et al., 2012; Hedderwick et al., 2017; Ariel de Lima et al., 2019; Atkinson et al., 2022). The methodology was repeated for the different knee joint ligaments. A more elaborate description of the process details was previously provided by Audenaert et al. and Peiffer et al. (Audenaert et al., 2019b; Peiffer et al., 2022a).

**TABLE 1:** The assigned ligament-specific mid-substance thickness (in millimeters), based on cadaveric studies and MRI measurements.

	MPFL	LPFL	sMCL	LCL	ALL	POL	OPL
Thickness (in mm)	2.90	1.80	2.10	2.20	1.50	1.00	1.44

**FIGURE 5:**
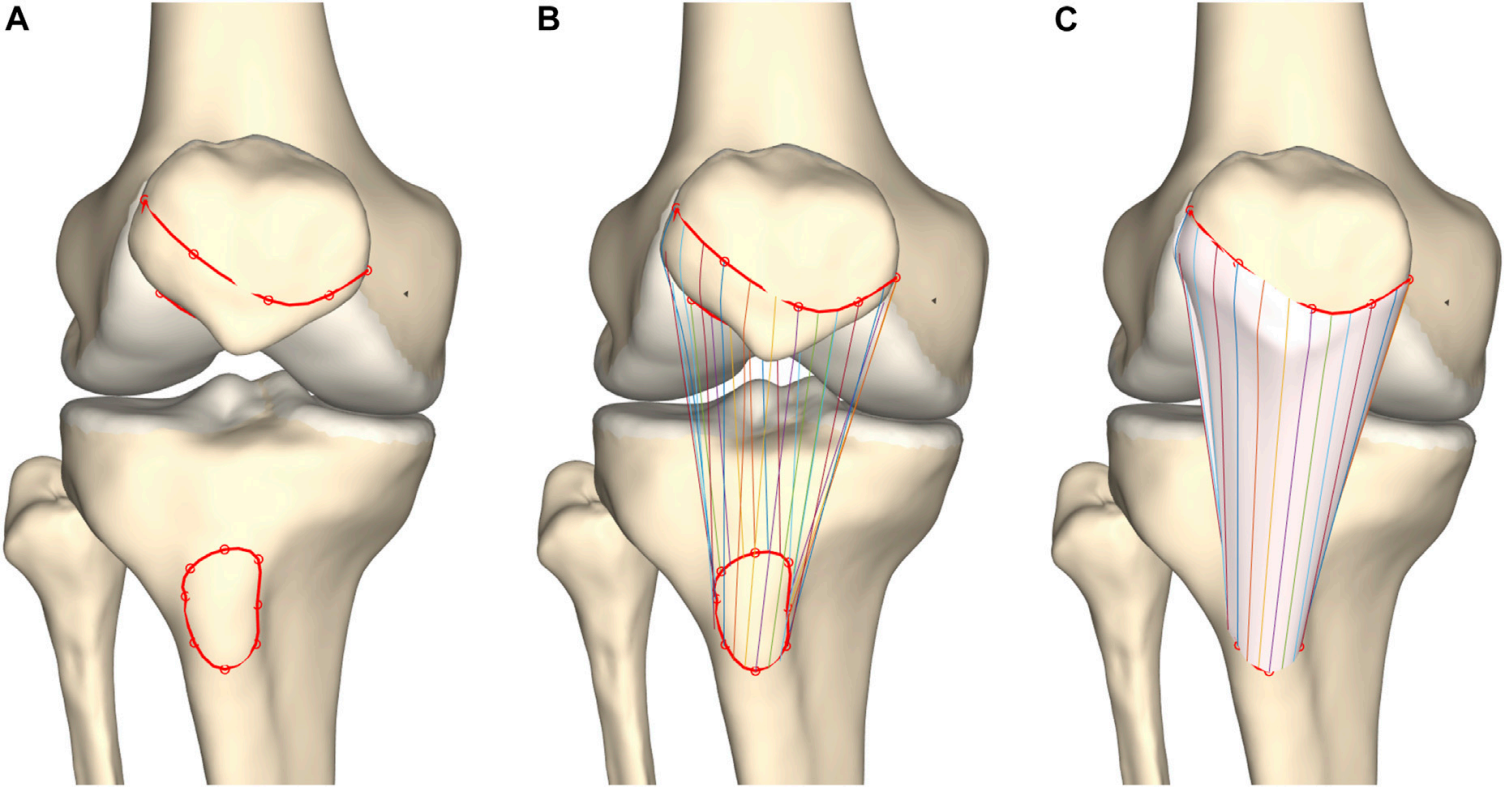
Frontal view of the knee joint. **(A)** Splines were formed interconnecting patellar origin and tibial insertion vertices. **(B)** Corresponding coordinates were connected. **(C)** Tendon modeling was based on a custom-made mesh wrapping algorithm.

Prediction and modeling of the patellar tendon is an exception on the above described technique. On both the origin and insertion site a spline was defined connecting the origin and insertion vertices respectively. Similarly as for ligamentous path prediction, corresponding coordinates were connected based on a custom-made mesh wrapping algorithm while any penetration of the combined osseous–cartilage–meniscus surface was corrected. As corresponding points on a closed spline were connected, the thickness of the ligament is inherent to the positioning of the vertices and is therefore not an assigned value (Figure 5).

#### 2.3.4 Cruciate ligament anatomy prediction

**FIGURE 6:**
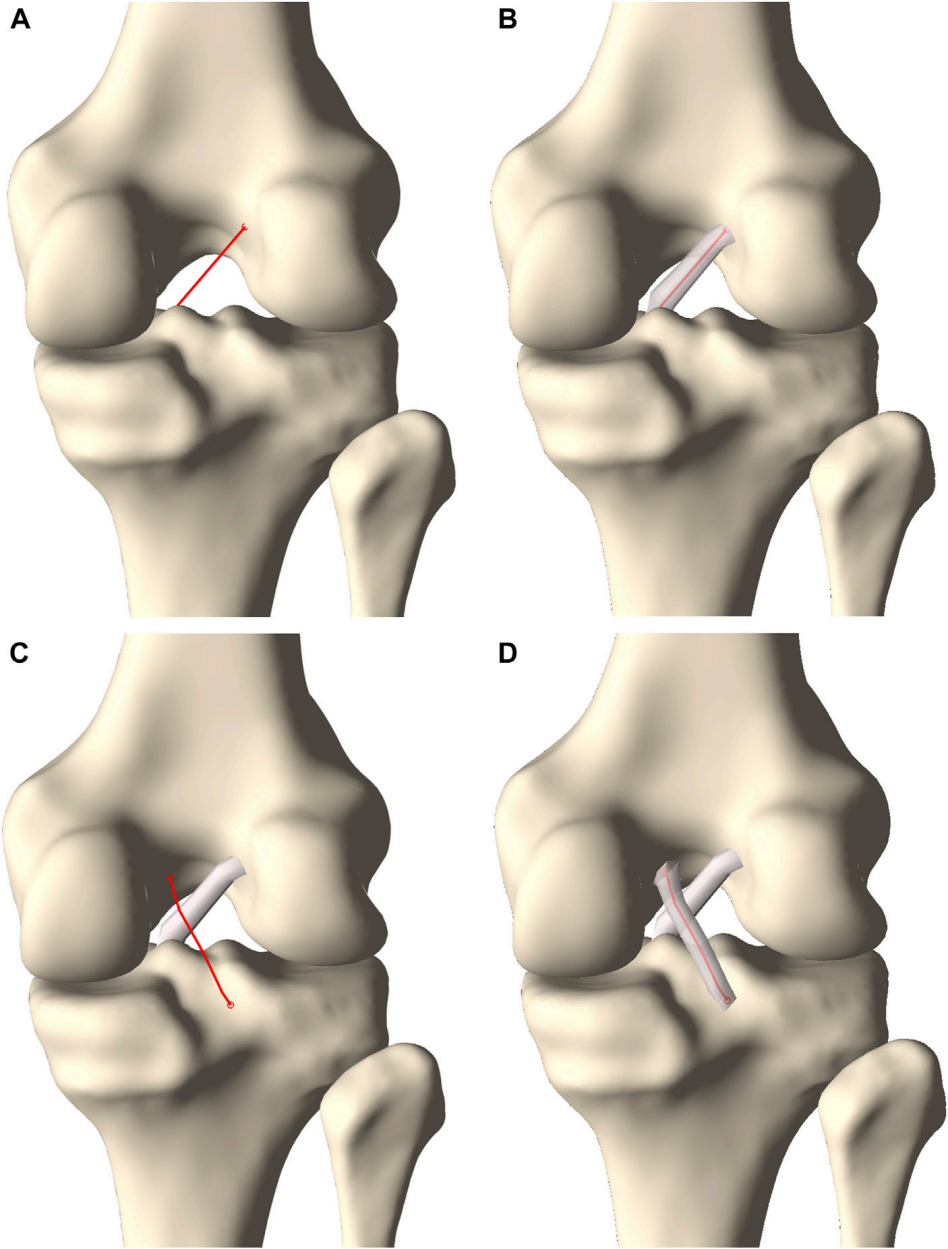
Posterior view on the knee. **(A)** A centerline was generated connecting the femoral ACL origin and the tibial ACL insertion. **(B)** A tube with varying radius was fitted around the centerline to model the ACL. **(C)** A second centerline was forced to wrap around the ACL when connecting the femoral PCL origin and the tibial PCL insertion. **(D)** A tube fitted around the centerline with a varying radius formed the PCL.

The cruciate ligaments were modeled as curved tubes with variable radii in three dimensional space, based on estimating Frenet-Serret frames along a centerline. First, the centerline of the ACL was modeled from origin to insertion as a series of connected spring elements (*n* = 15) and its was position optimized based on a shortest path function, similar as for initial ligament modeling (Audenaert et al., 2009; Audenaert et al., 2019b). Connecting nodes between spring elements were spatially constrained to a minimal offset similar to the cruciate ligament width. Second, and contrary to previous ligamentous modeling, regional variation in ACL thickness was appraised by low degree polynomial functions, the degree of which was determined following a similar sensitivity analysis as conducted for meniscal height and width. Third, the ACL was comprised in the osseous–cartilage–meniscus–ligament model to function as a constraint for the course of the PCL. The PCL was similarly modeled and thus wrapped around the ACL in its course from origin to insertion (Figure 6).

**FIGURE 7:**
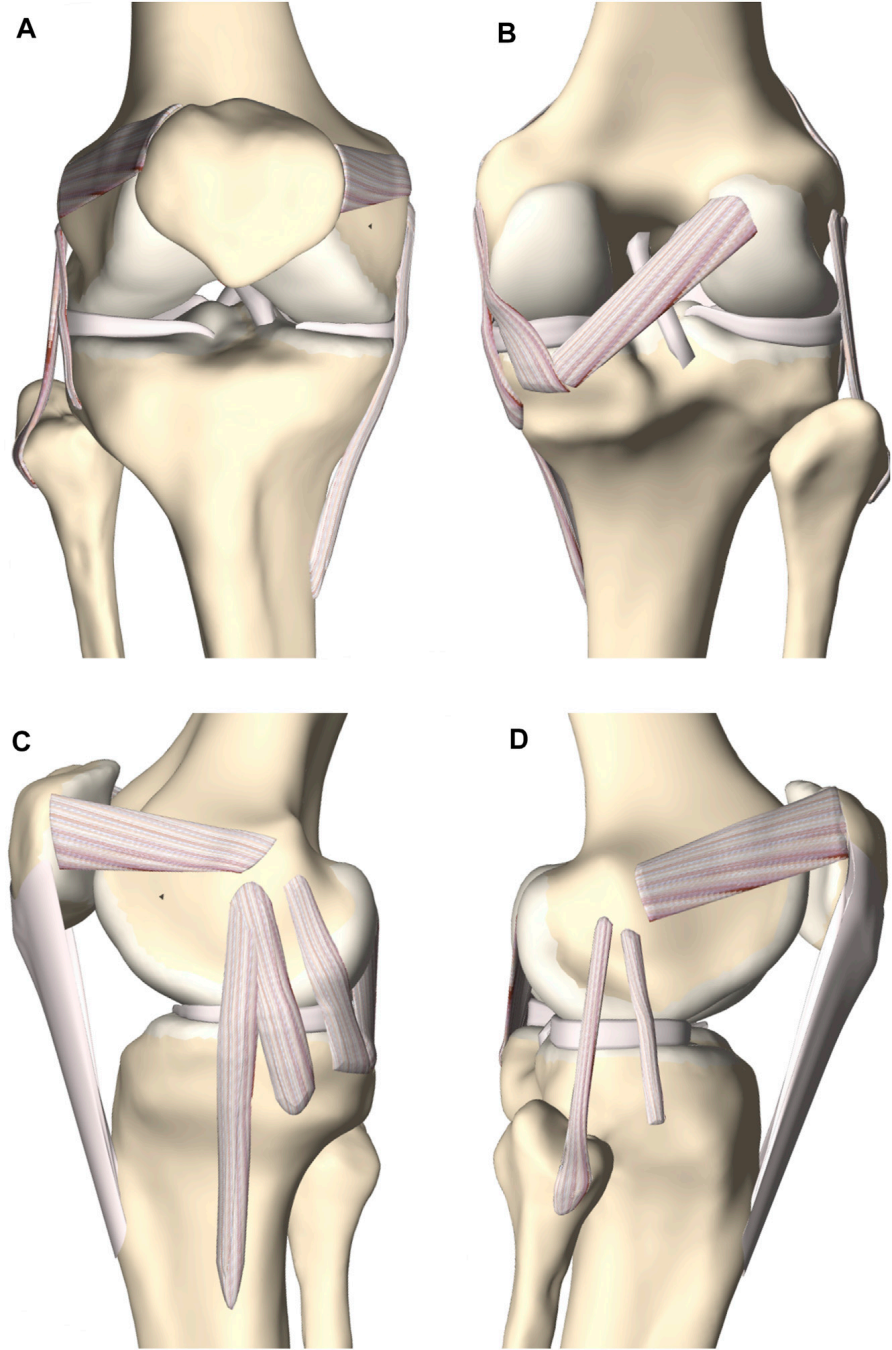
**(A)** Frontal, **(B)** posterior, **(C)** medial and **(D)** lateral view of the knee joint. The femoral, tibial and patellar bone were depicted including the predicted cartilage layer. The model contained the main knee ligaments, the patellar tendon (except in the frontal view), the medial and lateral meniscus and the anterior and posterior cruciate ligament.

A complete knee joint model was generated combining the modeled structures, described in section 2.3 (Figure 7).

### 2.4 Validation of soft tissue anatomy prediction

Validation of the predicted soft tissue anatomy relied on leave-one-out experiments. The predicted and manually detected anatomy was compared based on the Root Mean Square Error (RMSE, square root of the average of all absolute square distances), the Average Surface Distance (ASD, the average of all the distances) and the Hausdorff distance (HD, the maximum absolute distance).

#### 2.4.1 Validation of cartilage thickness prediction

For all the cases, the predicted and manually segmented node-specific cartilage thicknesses were compared (*n* = 53).

#### 2.4.2 Validation of ligament and patellar tendon anatomy prediction

##### 2.4.2.1 Validation of ligamentous and patellar tendon landmark identification

Manually selected ligament and tendon origin and insertion surface areas were compared with the predicted surface areas (*n* = 10). The surface areas were delineated as curves on the osseous structure. Curves were formed using a spline generating tool to interconnect both the manually selected and the predicted vertices on origin and insertion site.

##### 2.4.2.2 Validation of ligament and patellar tendon geometry prediction

Similar to the methodology described by Peiffer et al., the edges of the main knee ligaments and patellar tendon were manually identified on MR imaging (*n* = 10) (Peiffer et al., 2022b). A nearest neighbor algorithm was used to compare manually detected edges with the edges of the predicted course.

##### 2.4.2.3 Validation of cruciate ligament landmark identification

The manually selected origin and insertion of ACL and PCL were compared with the predicted origin and insertion (*n* = 10).

##### 2.4.2.4 Validation of cruciate ligament geometry prediction

The edges of the cruciate ligaments were manually determined on MR imaging and a nearest neighbor algorithm was used to compare with the predicted ligamentous edges (*n* = 10).

#### 2.4.3 Validation of meniscal anatomy prediction

##### 2.4.3.1 Validation of meniscal landmark identification

The anterior and posterior root of the medial and lateral meniscus were manually selected on MR imaging and compared with the predicted anterior and posterior root (*n* = 10).

##### 2.4.3.2 Validation of meniscal geometry prediction

**FIGURE 8:**
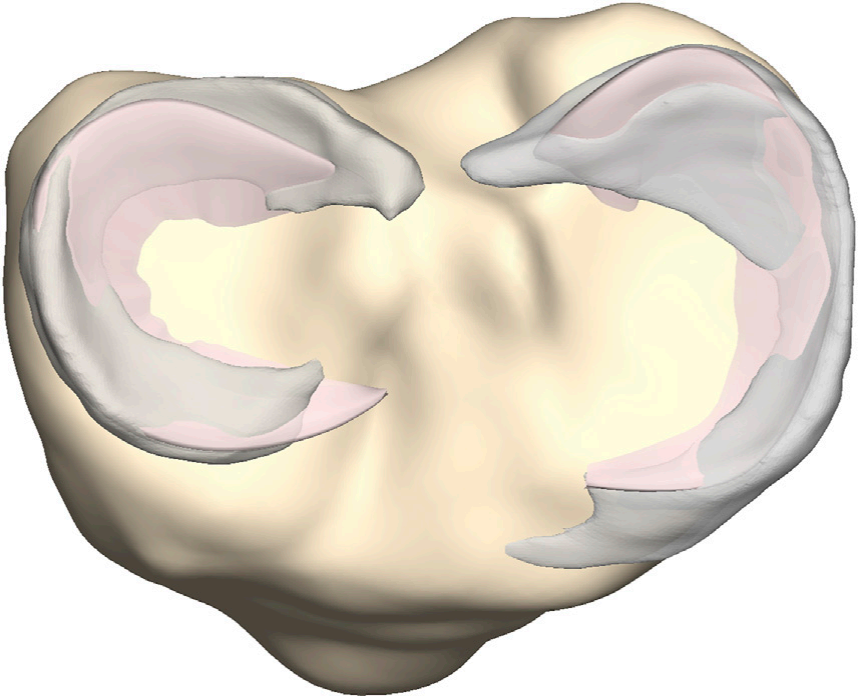
Axial view on the tibial plateau (yellow). The predicted medial and lateral menisci (pink) were superimposed on the manually segmented medial and lateral menisci (grey).

Manually segmented medial and lateral menisci, unseen to the model, were compared to the predicted medial and lateral meniscus (*n* = 10) (Figure 8). The point-dependent error was averaged over the cases and plotted to identify regions that contained most variation.

## 3 Results

### 3.1 Mean cartilage thickness maps

**FIGURE 9:**
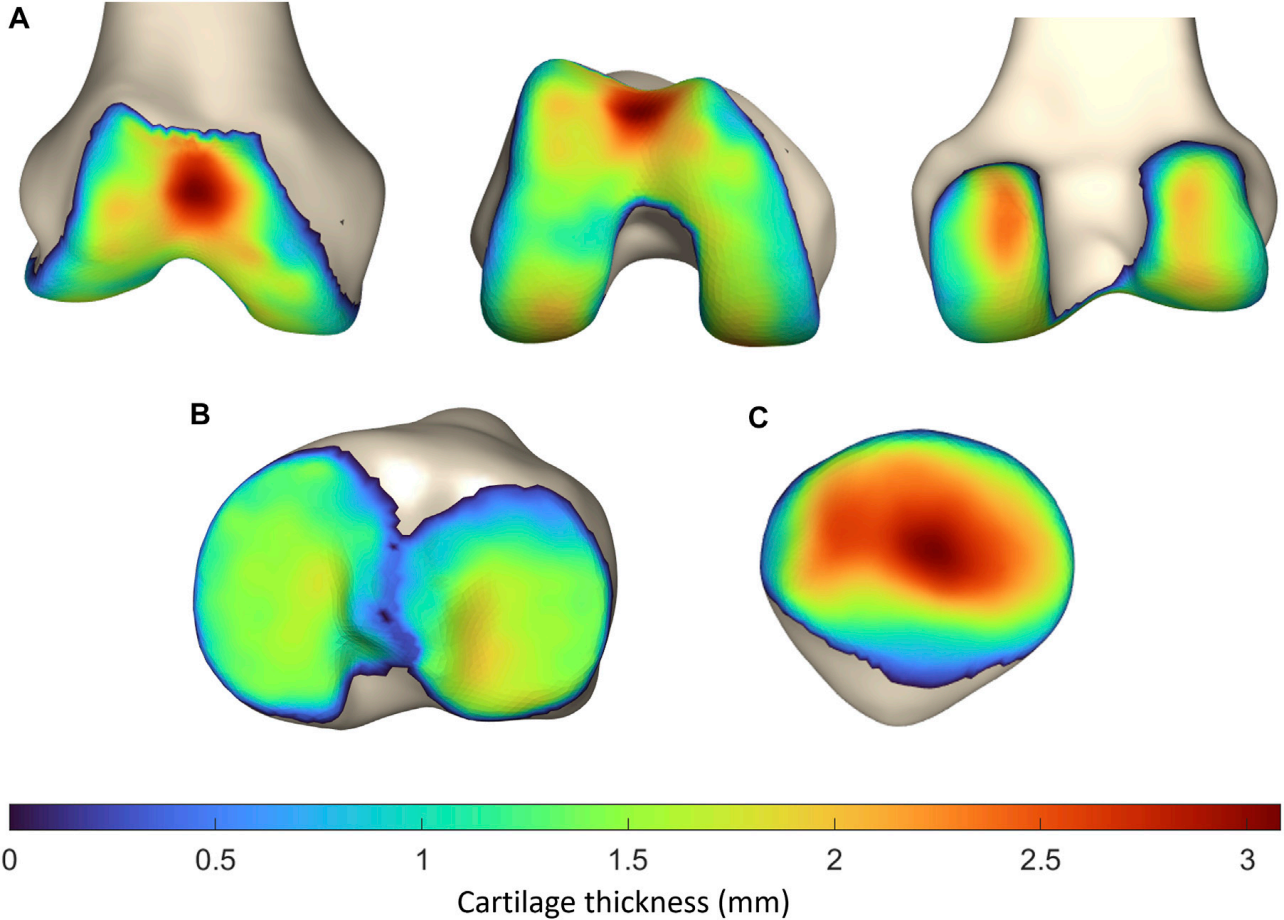
Representation of the vertex-specific cartilage thickness on **(A)** the mean femur, **(B)** the mean tibia and **(C)** the mean patella, extracted from the SSM. The average cartilage thickness ranges from 0 mm (blue) to 3.23 mm (red).

The average femoral cartilage thickness totaled 1.41 mm (SD 0.37, range 0–3.08 mm), with local cartilage thickness maxima located at the patellofemoral joint surface and the posterior condyles. Similarly, the average cartilage thickness equaled 1.10 mm (SD 0.31, range 0–1.71 mm) and 1.19 mm (SD 0.34, range 0–1.89 mm) for respectively the medial and lateral tibial plateau. For the patella, the average cartilage thickness was 1.69 mm (SD 0.72, range 0–3.23 mm). Local variation is shown in Figure 9.

### 3.2 Validation of soft tissue anatomy prediction

#### 3.2.1 Validation of cartilage thickness prediction

The largest error was observed for the prediction of the patellar cartilage thickness with a median RMSE of 0.75 mm (range 0.16–1.11 mm), a median ASD of 0.60 mm (range 0.13–0.89 mm) and a median HD of 2.05 mm (range 0.40–3.55 mm) (*n* = 53). Smaller errors and in the same order of magnitude were observed for prediction of tibial and femoral cartilage thickness prediction. The findings were summarized in Table 2.

**TABLE 2:** Median RMSE, ASD and HD with range for tibial, femoral and patellar cartilage layer prediction.

	Tibial cartilage	Tibial cartilage	Femoral cartilage	Patellar cartilage
	Medial plateau	Lateral plateau		
**RMSE (mm) (range)**	0.32 (0.14–0.48)	0.35 (0.16–0.53)	0.39 (0.15–0.80)	0.75 (0.16–1.11)
**ASD (mm) (range)**	0.26 (0.11–0.38)	0.29 (0.13–0.43)	0.31 (0.12–0.67)	0.60 (0.13–0.89)
**HD (mm) (range)**	0.80 (0.34–2.06)	0.90 (0.32–1.81)	0.96 (0.35–2.66)	2.05 (0.40–3.55)

**FIGURE 10:**
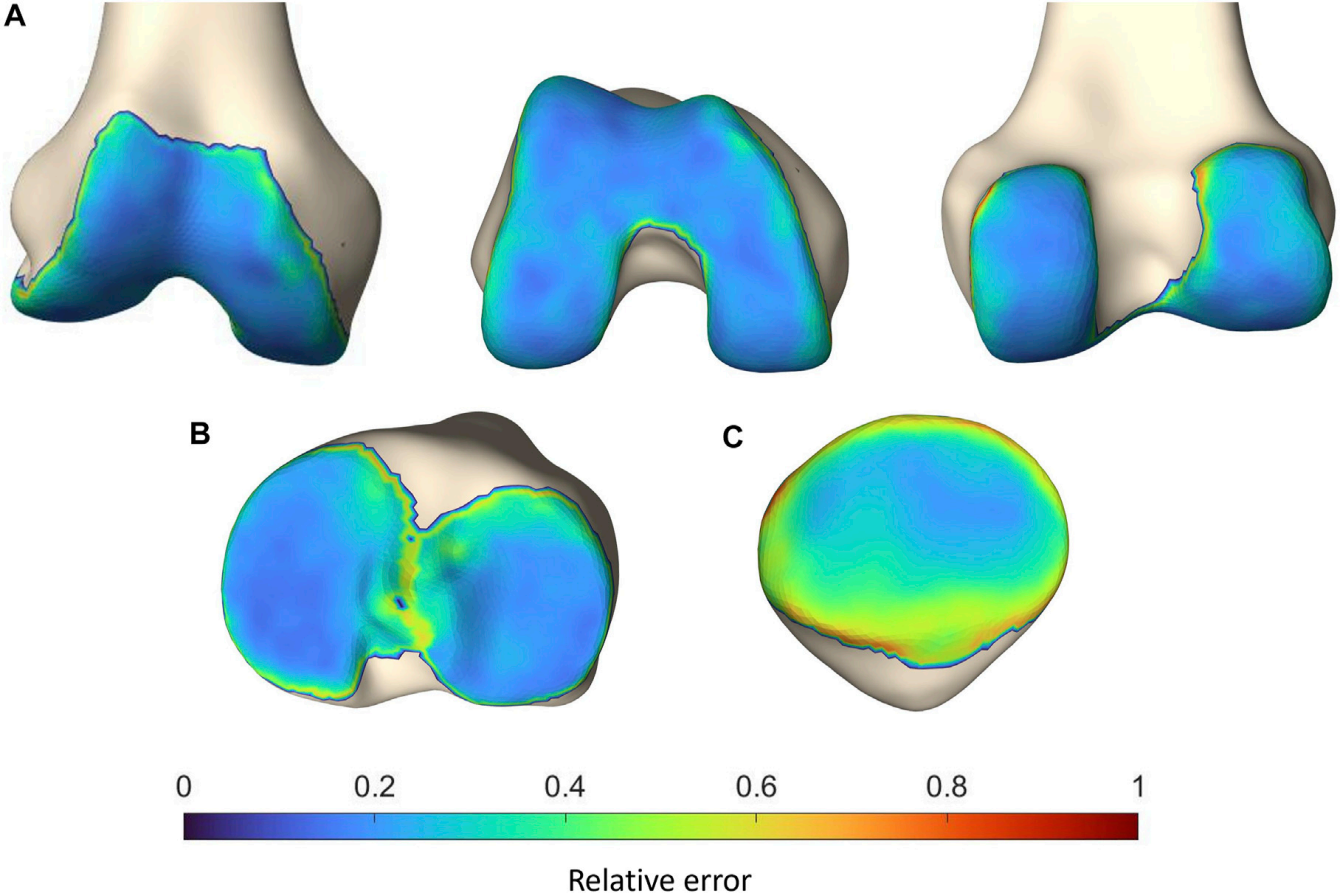
Representation of the vertex-specific relative error in cartilage thickness prediction on **(A)** the mean femur, **(B)** the mean tibia and **(C)** the mean patella, extracted from the SSM. Color-coding ranges from blue (absent error) to red (error equaling the point-dependent mean cartilage thickness).

To localize the sites with the largest error in cartilage thickness prediction, the point-dependent mean error is plotted relative to the point-dependent mean cartilage thickness (Figure 10).

#### 3.2.2 Validation of ligament and patellar tendon prediction

##### 3.2.2.1 Validation of ligamentous and patellar tendon landmark identification

Errors in predicting the osseous origin and insertion sites by landmark transfer were evaluated. The median RMSE ranged from 1.49 mm to 4.61 mm. The minimal and maximal error was described respectively for the femoral origin of the LCL and the tibial insertion of the anterior bundle of the sMCL. Detailed findings are presented in Table 3.

**TABLE 3:** Median RMSE, ASD and HD with range for the main knee joint ligaments and patellar tendon origin and insertion.

Origin	MPFL (patella)	LPFL (patella)	sMCL anterior bundle (femur)	sMCL posterior bundle (femur)	LCL (femur)	ALL (femur)	POL (femur)	OPL (femur)	PT (patella)
**RMSE (in mm) (range)**	2.82 (0.35–5.16)	3.06 (2.92–7.12)	2.08 (1.05–4.14)	2.22 (1.00–3.93)	1.49 (1.17–2.78)	3.71 (3.63–4.61)	2.66 (1.12–7.37)	2.71 (2.03–4.69)	2.78 (0.86–4.39)
**ASD (in mm) (range)**	1.84 (0.20–4.79)	2.90 (2.35–5.87)	1.92 (0.70–4.09)	2.04 (0.72–3.52)	1.41 (1.13–2.61)	2.61 (2.02–2.70)	2.49 (1.03–7.33)	1.90 (1.49–4.31)	2.73 (0.77–3.76)
**HD (in mm) (range)**	6.44 (1.31–9.87)	5.94 (5.41–12.33)	3.86 (2.22–7.62)	3.59 (2.14–5.62)	1.89 (1.81–3.47)	12.18 (11.33–13.88)	3.51 (1.89–8.05)	7.57 (4.78–9.51)	4.17 (1.81–7.62)

Insertion	MPFL (femur)	LPFL (femur)	sMCL anterior bundle (tibia)	sMCL posterior bundle (tibia)	LCL (fibula)	ALL (tibia)	POL (tibia)	OPL (tibia)	PT (tibia)
**RMSE (mm) (range)**	2.82 (2.37–5.42)	4.44 (4.13–6.88)	4.61 (2.71–7.08)	3.22 (2.65–13.36)	1.57 (0.16–2.91)	2.49 (1.84–3.11)	1.72 (1.66–3.01)	1.63 (1.36–3.24)	2.67 (0.79–6.06)
**ASD (in mm) (range)**	2.37 (1.74–4.46)	4.42 (3.53–6.03)	3.49 (1.88–5.28)	2.56 (2.23–13.15)	1.45 (0.14–3.48)	2.39 (1.63–3.05)	1.55 (1.03–2.45)	1.46 (1.15–2.96)	1.80 (0.67–4.96)
**HD (in mm) (range)**	5.95 (3.84–10.14)	6.86 (5.04–10.53)	10.05 (6.21–15.10)	5.83 (4.76–15.10)	2.41 (0.23–3.48)	3.33 (2.65–4.12)	4.67 (3.25–6.57)	2.90 (2.49–5.53)	5.26 (1.44–9.56)

##### 3.2.2.2 Validation of ligament and patellar tendon geometry prediction

The median RMSE in predicting the shape of the ligaments and patellar tendon ranged from 0.27 mm to 1.04 mm. The largest variation was observed in patellar tendon prediction with a median ASD of 0.80 mm (range 0.74–0.97 mm) and a median HD of 2.20 mm (range 1.64–2.84 mm). The smallest error was observed for the POL with a median ASD of 0.23 mm (range 0.14–0.36 mm) and a median HD of 0.49 (range 0.29–1.07). These and additional findings are summarized in Table 4.

**TABLE 4:** Median RMSE, ASD and HD with range for the main knee joint ligaments and the patellar tendon course.

	MPFL	LPFL	sMCL anterior bundle	sMCL posterior bundle	LCL	ALL	POL	OPL	PT
**RMSE (in mm) (range)**	0.46 (0.24–1.05)	0.34 (0.19–0.57)	0.44 (0.26–0.59)	0.40 (0.37–0.53)	0.47 (0.27–1.29)	0.51 (0.40–0.91)	0.27 (0.17–0.47)	0.43 (0.24–0.72)	1.04 (0.85–1.13)
**ASD (in mm) (range)**	0.35 (0.23–0.84)	0.29 (0.17–0.40)	0.38 (0.25–0.57)	0.34 (0.30–0.44)	0.40 (0.25–1.03)	0.43 (0.34–0.68)	0.23 (0.14–0.36)	0.38 (0.21–0.57)	0.80 (0.74–0.97)
**HD (in mm) (range)**	0.60 (0.34–1.63)	0.69 (0.31–1.64)	0.80 (0.39–1.12)	0.75 (0.65–0.98)	0.79 (0.35–2.63)	0.70 (0.58–1.70)	0.49 (0.29–1.07)	0.70 (0.42–1.48)	2.20 (1.64–2.84)

##### 3.2.2.3 Validation of cruciate ligament landmark identification

The largest error in predicting the osseous origin and insertion was observed for the tibial insertion of the ACL. The RMSE equaled 6.29 mm and the median error equaled respectively 5.65 mm (range 4.15–8.66 mm). Additional findings are summarized in Table 5.

**TABLE 5:** The RMSE and the median error with range from minimal to maximal error for the ACL and the PCL origin and insertion.

Origin	ACL (femur)	PCL (femur)	Insertion	ACL (tibia)	PCL (tibia)
**RMSE (in mm)**	2.70	2.25	**RMSE (in mm)**	6.29	2.97
**Error (in mm) (range)**	2.02 (0–3.84)	2.54 (0–3.14)	**Error (in mm) (range)**	5.65 (4.15–8.66)	2.69 (0–4.83)

##### 3.2.2.4 Validation of cruciate ligament geometry prediction

Only small errors were observed in predicting cruciate ligament geometry. The RMSE equaled 1.16 mm (range 0.99–1.59) and 0.91 mm (range 0.75–1.33) for respectively the PCL (Table 6).

**TABLE 6:** Median RMSE, ASD and HD with range for the ACL and PCL course.

	ACL	PCL
**RMSE (in mm) (range)**	1.16 (0.99–1.59)	0.91 (0.75–1.33)
**ASD (in mm) (range)**	1.15 (0.89–1.39)	0.87 (0.72–1.26)
**HD (in mm) (range)**	1.60 (1.41–2.61)	1.35 (0.95–2.15)

**FIGURE 11:**
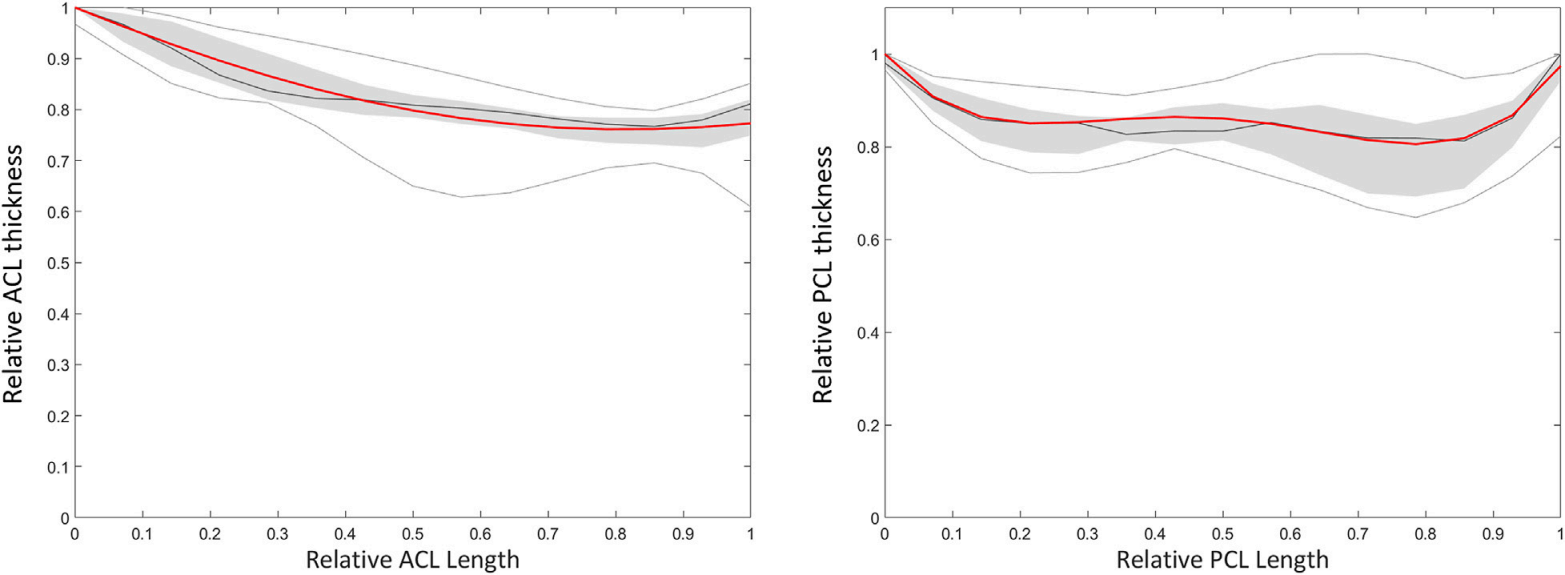
Polynomial fitting for ACL and PCL thickness prediction. The median thickness of the ACL (left) and PCL (right) is presented as the dark grey line. The grey zone represents the values between P25 and P75. The light grey lines represent minimal and maximal values. The red line represents the thickness based on a respectively 2nd and 4th degree polynomial function for the ACL and PCL.

Based on a sensitivity study the degree of polynomial fitting for prediction of local variation in thicknesses from origin to insertion for both the ACL and the PCL was determined. The optimal polynomial fit was respectively a 2nd and 4th degree polynomial fit (Figure 11). The measured and predicted cruciate thickness was compared for validation purposes (Table 7).

**TABLE 7:** Median RMSE, ASD and HD with range between predicted and measured thickness of the ACL and PCL.

	ACL	PCL
**RMSE (mm) (range)**	0.17 (0.06–0.48)	0.95 (0.64–1.42)
**ASD (mm) (range)**	0.14 (0.05–0.35)	0.88 (0.58–1.30)
**HD (mm) (range)**	0.27 (0.11–0.94)	1.36 (0.94–1.99)

#### 3.2.3 Validation of meniscal anatomy prediction

##### 3.2.3.1 Validation of meniscal landmark identification

The median RMSE in landmark prediction ranged from 2.71 mm to 3.75 mm. In general, a larger error was observed for prediction of the location of the anterior root in comparison to the prediction of the location of the posterior root. Detailed findings are described in Table 8.

**TABLE 8:** The RMSE and the median error with range from minimal to maximal error for medial and lateral meniscus anterior and posterior root.

Anterior root	Medial meniscus	Lateral meniscus	Posterior root	Medial meniscus	Lateral meniscus
**RMSE (in mm)**	3.51	3.75	**RMSE (in mm)**	2.71	2.82
**Error (in mm) (range)**	3.31 (1.37–5.10)	2.91 (1.49–5.29)	**Error (in mm) (range)**	1.78 (1.47–5.22)	2.45 (0–4.22)

##### 3.2.3.2 Validation of meniscal geometry prediction

**FIGURE 12:**
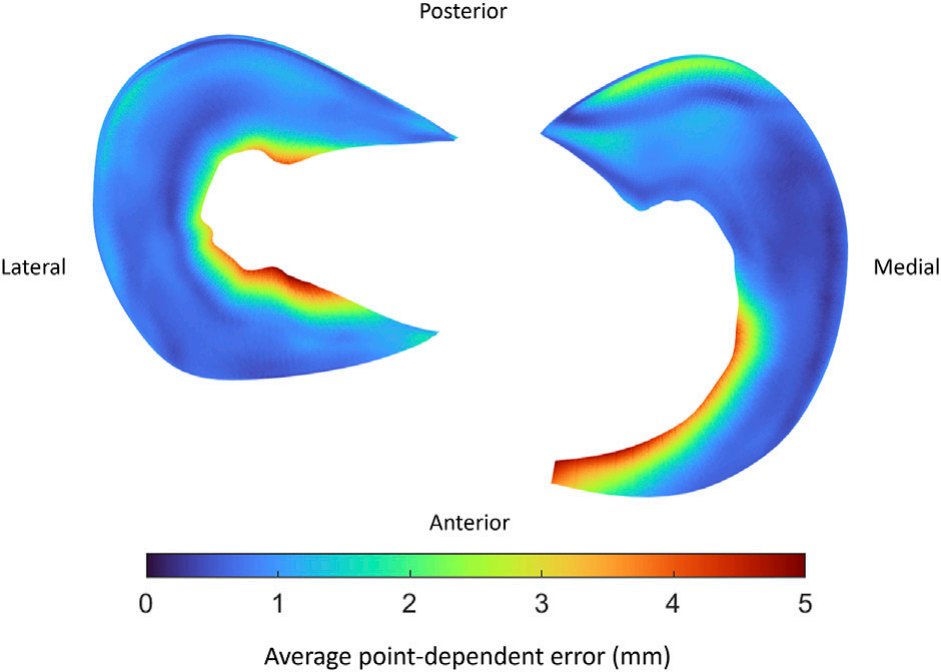
Visual representation of the average point-dependent error for the mean predicted lateral (left) and medial (right) meniscus. The mean error was color-coded ranging from blue towards red, equaling respectively an average point-dependent error of 0 mm and 5 mm.

Comparable errors were observed for the medial and lateral meniscus. The median RMSE equaled respectively 2.93 mm (range 1.85–4.66) and 2.04 mm (range 1.88–3.29) (Table 9). The average point-dependent error ranged from 0 mm to 5 mm. The local variation in meniscal geometry prediction was largest for the inner rim of the lateral meniscus and the anterior root of the medial meniscus. The error was plotted in Figure 12.

**TABLE 9:** Median RMSE, ASD and HD with range for the medial and lateral meniscal course.

	Medial meniscus	Lateral meniscus
RMSE (range)	2.93 (1.85–4.66)	2.04 (1.88–3.29)
ASD (range)	1.84 (1.21–3.26)	1.49 (1.30–2.40)
HD (range)	11.64 (7.97–17.09)	9.79 (6.09–14.43)

**FIGURE 13:**
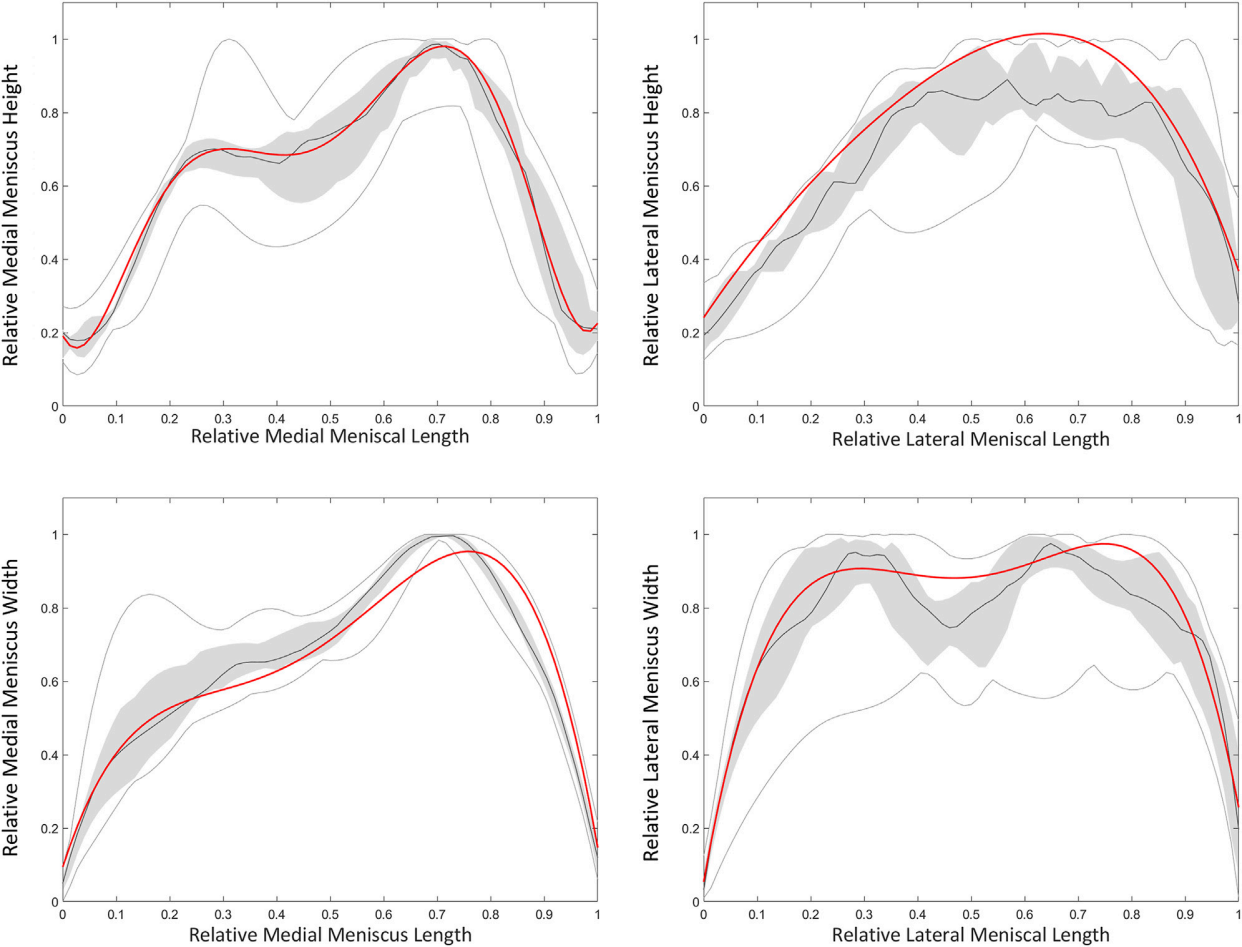
Polynomial fitting for medial and lateral meniscal height and width prediction. The median height and width of the medial (left) and lateral (right) meniscus is presented as the dark grey line. The grey zone represents the values between P25 and P75. The light grey lines represent minimal and maximal values. The red line represents height and width of respectively a 6th and 4th degree polynomial function for the medial meniscus. For the lateral meniscus, the red line represents twice a 4th degree polynomial fit.

Based on a sensitivity study the degree of polynomial fitting for prediction of meniscal height and width was determined. For the medial meniscus, height and width were plotted as respectively a 6th and 4th degree polynomial function. For the lateral meniscus, height and width were both plotted as a 4th degree polynomial function (Figure 13). The degree of polynomial fitting was validated comparing the measured and predicted meniscal height and width (Table 10).

**TABLE 10:** Median RMSE, ASD and HD with range between predicted and measured height and width of the medial meniscus (MM) and lateral meniscus (LM).

	Medial meniscus (height)	Lateral meniscus (height)	Medial meniscus (width)	Lateral meniscus (width)
RMSE (mm) (range)	3.88 (1.18–6.43)	2.96 (1.49–3.75)	2.77 (1.51–3.69)	2.27 (1.04–5.41)
ASD (mm) (range)	3.71 (0.97–6.06)	2.45 (1.17–3.27)	2.40 (1.27–3.20)	1.97 (0.89–4.28)
HD (mm) (range)	5.97 (2.44–9.47)	5.55 (3.63–7.12)	4.94 (2.68–7.33)	4.18 (1.91–10.78)

## 4 Discussion

We present a methodological workflow for the development of a morphological knee model based on individual osseous morphology to automate the prediction of soft tissue anatomy. Computational models are on the rise for techniques to estimate joint kinetics, however soft tissue inclusion generally depends on laborious manual segmentation. For example, Dong et al. developed a three-dimensional knee joint model combining osseous elements with cartilage layers, cruciate and collateral ligaments, menisci and tendon insertions, solely based on manual segmentations (Dong et al., 2014). Similarly, Kang et al. generated a three-dimensional knee joint model in the development process of a finite element model to assess weight-bearing related deformation of the intra-articular cartilage contact area (Kang et al., 2015). As knee joint malalignment contributes to the onset and progression of joint OA, Mootanah et al. predicted knee joint contact forces and pressures depending on the amount of varus-valgus malalignment (Mootanah et al., 2014). Non-etheless, the model generation is very time-consuming and generalization to other cases and patient geometries is not straightforward.

Aiming to avoid manual segmentation and allow for patient specific analysis, we predict soft tissue anatomy relying solely on the underlying osseous morphology and capitalizing on the advantage of point correspondence and uniformly, isometrically distributed meshes when using SSM (Dong et al., 2014; Mootanah et al., 2014; Kang et al., 2015; Audenaert et al., 2019a). As a result, the extensive workload related to manual segmentation to obtain patient specific description of bony and soft tissue can be avoided (Audenaert et al., 2019a). Furthermore, as all computations originate from skeletal statistical shape modeling, and considering the generative power of these SSMs, large virtual cohorts can be defined for population-wide studies.

In analogy with a mesh node-specific cartilage thickness was allocated and averaged over the cohort in the development of a mean cartilage thickness map (van Houcke et al., 2020a). Previous research has already demonstrated the correlation between osseous size and cartilage layer thickness (van Dijck et al., 2018; Schneider et al., 2022). Proven to be an accurate estimator for total body length, we scaled the mean distance map by the femoral length (Hauser et al., 2005). To avoid small errors in underlying skeletal anatomy, possibly introduced by SSM-based automated image segmentation, to influence the validation process of cartilage thickness prediction, we compared the scaled, mean distance map to the case-specific calculated distance map instead of comparing the predicted and segmented cartilage surfaces (Seim et al., 2008; Bae et al., 2009). Of note, a small amount of variation in total length of the investigated population was observed, with a 95% confidence interval ranging from 180.1 to 183.5 cm. As a result, little variation was introduced in the imposed scaling factor.

The observed RMSE and ASD for femoral and tibial cartilage prediction was smaller than the MRI pixel size of 0.469 mm, on which our methodology was based. Although the prediction error for the patellar cartilage layer was slightly larger than the pixel size, it did not exceed 1.0 mm. Furthermore, when comparing to manual cartilage segmentation, considered the ground truth, the observed RMSE and ASD are in the same order of magnitude of previously published results. Manual segmentation itself is thus subject to intra- and inter-observer variation, inherent to the manual aspect of the technique. Part of the reported error in this study is therefore attributable to error related to manual segmentation (Cohen et al., 1999).

Since a relatively large HD of 2.05 mm was observed for patellar cartilage layer prediction, the zones with the largest errors were identified. Therefore, the obtained point-dependent mean error was then evaluated relative to the point-dependent mean cartilage thickness. The zones with the relatively largest local error are located near the edges of the cartilage layers and are rather small. Cartilage thickness prediction at the weight bearing sites, on the other hand, present a relatively small absolute error of approximately 0.6 mm, 0.36 mm and 0.6 mm for respectively the femoral bone, the tibial plateau and the patellar bone. In the absence of complete knee joint DEA modeling, the influence of cartilage thickness errors was evaluated for DEA modeling of the hip joint based on the available research. Niknafs and colleagues compared the relative decrease in peak contact stresses based on DEA modeling for different types of cartilage modeling following alignment optimization. For an acetabular cartilage thickness ranging between 1.24 and 1.95 mm a relative decrease of 39% was observed. For increased cartilage thickness, ranging from 1.29 mm to 2.87 mm, a greater relative decrease of 47% was observed. Thus, a thicker cartilage layer results in decreased peak stresses based on DEA modeling. Future research is however necessary to evaluate the impact of the variation in cartilage thickness on DEA-based stress calculations in the knee joint (Cohen et al., 1999).

**FIGURE 14:**
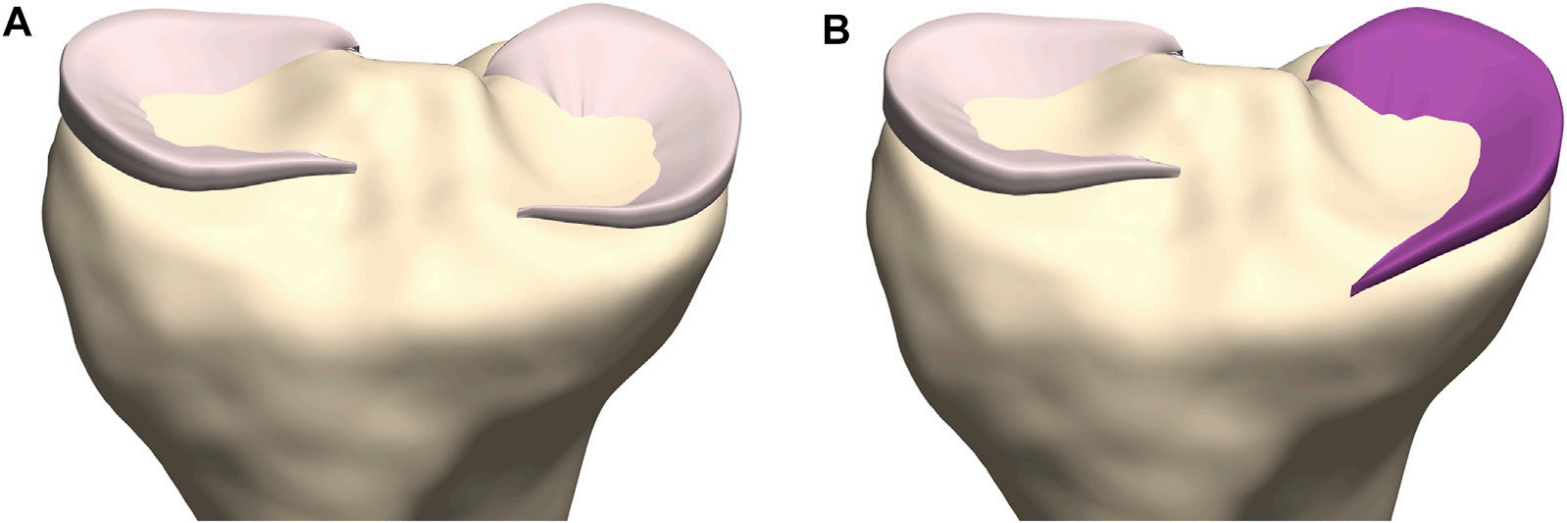
Coronal view on the tibial plateau (yellow). **(A)** Prediction of the medial meniscus according to the developed workflow demonstrates the type I insertion as described by De Coninck and colleagues. **(B)** By changing the anterior root insertion reference, a medial meniscus with a type III insertion is modeled (Mootanah et al., 2014).

Subsequently, we predicted meniscal anatomy as mobile, elastic structures connecting origin and insertion and being able to accommodate to the shape of the femoral condyles and their variable position relative to the tibia. As such, meniscal anatomy prediction was based on the underlying osseous geometry. Vrancken and colleagues manually segmented the medial meniscus with a 6 months’ time period in between to evaluate the inter- and intra-observer variability. They report a relatively small RMSE of 0.29 and 0.27 for respectively the intra- and inter-observer reliability, in comparison with the reported errors in this study (Vrancken et al., 2014). However, the described error in meniscal course prediction when compared to manual segmentation is in the same order of magnitude as the findings of Tack and colleagues, who augmented their segmentation technique with the introduction of convolutional neural networks (Tack et al., 2018). Regarding meniscal origin and insertion prediction, we observe the largest prediction error to be present in the anterior root of the medial meniscus. This greater variation in anterior root error is explained as one out of the ten cases presents an extremely anterior insertion of the root. Variations in anterior root insertion of the medial meniscus are previously investigated by De Coninck and colleagues who identified three different bony insertion types on MRI. One of these types is described as an insertion anteriorly of the anterior tibial edge and is present in approximately 7% of the studied population (de Coninck et al., 2017). This specific anatomical variant was present in only one case of the cohort (*n* = 10) used for development of the meniscal model. The increased mean error positioned at the anterior root of the medial meniscus in the leave-one-out experiment can in part be attributed to this morphological variant. While currently not included in the present model, introduction of this variant in a probabilistic sense, is possible by simply changing the anterior root insertion reference (Figure 14). In large virtual population studies, different types of anterior root insertions can be included with the probability of a type I, II or III insertion linked to their respective population-wide prevalence.

Lastly, we included the cruciate ligaments, the main knee ligaments and the patellar tendon, again aiming to avoid manual segmentation and based on a fixed origin and insertion. To assess the accuracy of the main and cruciate ligamentous origin and insertion prediction, the described errors are evaluated against the available literature. Van der Merwe and colleagues reported MRI-based intra-observer agreements for manual femoral and tibial landmark identification. The intra-observer agreements ranged between 0.09 mm and 2.46 mm and between 0.06 mm and 2.69 mm for respectively the femoral and tibial bone (van der Merwe et al., 2019). Similarly, Esfandiarpour et al. studied the variability in landmark identification on the femoral condyles. The standard error of measurement for medial and lateral epicondyle landmark identification ranged from 0.41 mm to 0.78 mm, from 1.35 mm to 3.43 mm and from 1.03 mm to 4.71 mm in respectively the mediolateral, the craniocaudal and the anteroposterior direction (Esfandiarpour et al., 2009). The observed errors in this study are in the same order of magnitude as the previously described variability in manual landmark identification. Furthermore, for future application in DEA modeling, the general direction of the ligamentous fibers is of greater importance than absolute errors in origin and insertion identification. For a ligamentous length of 5 cm, a 3 mm error changes the orientation of the ligament with only 3.4°. Thus, in the case of a prediction error in the order of magnitude of the reported average errors, the fiber orientation will change only minimally. However, we have to acknowledge that for greater prediction errors, as reported by the Hausdorff distance, changes in fiber orientation up to 20° are currently inevitable. The exact impact of larger changes in ligamentous orientation for DEA-based stress calculations in the knee joint is yet to be examined (Peña et al., 2006).

Besides ligament origin and insertion, ligamentous thickness over the course is imposed based on measurements from cadaveric studies, with the exception of the thickness of the cruciate ligaments. However, since soft tissue contains a substantial amount of water, measurements based on cadaveric specimens are possibly influenced by tissue dehydration (Haut et al., 1998). For the prediction of cruciate ligament geometry, a varying thickness is imposed based on MRI measurements. This matches the cadaveric findings of Triantafyllidi and colleagues, who described the thickness of the ACL varying from a small femoral attachment towards a broad tibial attachment whereas the PCL showed a large femoral and tibial attachment with a smaller mid-substance (Triantafyllidi et al., 2013). However, accurate assessment of ligamentous thickness and osseous insertions based on MRI imaging is challenging (Rachmat et al., 2014). Based on the results of the validation experiments, we are able to predict cruciate ligaments, main knee ligament and patellar tendon anatomy with a large accuracy.

### 4.1 Strengths

We present a novel methodology for personalized, static knee joint modelling, avoiding laborious manual segmentation tasks and improving generalizability. Being less time-consuming, it allows for easier applicability in a clinical setting. Starting from CT or MRI imaging, the osseous anatomy of the individual patient can easily be extracted, requiring only a few minutes to manually allocate the osseous edges on imaging data. Following the presented methodological pipeline, a comprehensive patient-specific knee joint model is available in less than 10 min, while avoiding additional manual interactions. However, with emerging applications in machine learning, the process of annotation and landmark identification can theoretically be performed real-time. The translation to the routine clinical practice by adopting these techniques will be the focus of future work. For example, accurate anatomical models enable generation of patient-specific instrumentation (PSI), recently introduced in total knee arthroplasty (TKA) (Watters et al., 2011; Reimann et al., 2019). Schotanus and colleagues demonstrated a superior outcome in terms of component alignment accuracy when relying on MRI-based PSI in comparing to CT- or X-ray-based PSI’s. This was most possibly a result of considering the cartilage layer in PSI development (Schotanus et al., 2016). Avoiding manual cartilage segmentation, Van Dijck and colleagues developed a statistical shape modeling based tibiofemoral cartilage prediction tool. Based on the reported RMSE and inter-observer variability, they proved to outperform manual segmentation accuracy. As such, accurate soft tissue predictions enable to develop PSI’s with an accuracy equaling MRI-based PSI’s while avoiding time-consuming manual soft tissue segmentation and MRI related high costs (van Dijck et al., 2018).

Outside the clinical field of orthopaedics, computational modeling of patient-specific knee joint morphology can function as an input for Discrete Element Analysis (DEA). DEA can be used for non-invasive estimation of intra-articular joint contact stress (van Houcke et al., 2020a; Peiffer et al., 2022b). The cornerstone, however, remains accurate, and preferably individualized, inclusion of soft tissue and osseous morphometrics. The extraction of detailed morphometrics from MRI imaging relies on one of the three segmentation methods, namely, manual, semiautomatic and automatic. However, no numerical data are available comparing knee joint DEA modeling based on manual segmentation to (semi-)automatic segmentation. Previous research has proven the time consuming aspect and the possibly large variability related to manual segmentation. Furthermore, the robustness and reproducibility of (semi-)automatic segmentation enables a more efficient introduction of DEA modeling in clinics (Heye et al., 2013). Therefore, differences in DEA-based intra-articular stress predictions attributable to the type of underlying mesh are subject for future research.

### 4.2 Limitations

An important limitation is the inclusion of a homogeneous population of western European descent in our study. Extrapolation to other populations is not advised since the complex interaction between genes, culture and the environment results in a population-based variation of morphological features. Even more, our findings are based on a group of men, aged between 17 and 25 years with a total body length of 95% of the individuals ranging between 180.1–183.5 cm. However we do not question the validity of the model, more subjects should be included in future research to expand the range of total body length of the investigated population. Moreover, inclusion of additional subjects would allow to collect supplementary metadata, such as activity level and body mass index which are often discussed risk factors for cartilage loss, but were unfortunately not collected for the current dataset (Schneider et al., 2022).

Regarding meniscal inclusion, the menisci are solely statically modeled. Since MRI scanning was performed with the knee in fully extended position, uniquely validation of the meniscal position in a non-weight bearing, fully extended position was possible. As we hypothesized the menisci to adjust to the edges of the combined osseous–cartilage structure during stance, this concept could possibly be extrapolated to predict meniscal position during knee flexion. Improved insight in meniscal movement and validation of this concept requires currently lacking, additional MRI scanning in different degrees of knee flexion and should be subject to future research. However, modeling the menisci as an elastic tube from a fixed anterior to posterior root with varying height and subsequent allocation of a varying width allows for effortless translation towards meniscal modeling in different degrees of knee flexion.

Lastly, statistical modeling of soft tissue remains an approximation of reality and is based on a limited amount of samples to provide for the anatomical input in model development (*n* = 10). The aim is to find the optimal balance between including sufficient anatomical details on one hand and preserving computational efficiency on the other hand. As the reality is inevitably simplified, there is a definite need for validation tests to ensure that an adequate approximation is achieved. Furthermore, the pixel size of the used MR images influences the accuracy of anatomical landmark identification. Non-etheless, the described error range in this study is comparable to the relatively large inter- and intra-observer variability related to MRI-based manual soft tissue segmentations (Esfandiarpour et al., 2009; van der Merwe et al., 2019).

## 5 Conclusion

In conclusion, we present an innovative methodological workflow for personalized, static knee joint modelling. As soft tissue prediction is based on the underlying anatomy, we avoid laborious manual segmentation and allow for fast and personalized geometry predictions and maintain an accuracy comparable to manual soft tissue segmentation. Future research is necessary to implement an accurate morphometric knee joint model including the dynamic meniscus into clinical practice and thereby improve patient specific models of knee surgeries.

## Data Availability

The processed imaging data used for the model development in the manuscript are available upon request. Requests to access these datasets should be directed to emmanuel.audenaert@ugent.be. The original contributions presented in the study are publicly available. This data can be found here: https://www.mathworks.com/matlabcentral/fileexchange/124301-personalized-statistical-modeling-of-knee-soft-tissue.
